# Evidence for lysine acetylation in the coat protein of a polerovirus

**DOI:** 10.1099/vir.0.066514-0

**Published:** 2014-10

**Authors:** Michelle Cilia, Richard Johnson, Michelle Sweeney, Stacy L. DeBlasio, James E. Bruce, Michael J. MacCoss, Stewart M. Gray

**Affiliations:** 1 USDA-Agricultural Research Service, Ithaca, NY 14853, USA; 2 Department of Plant Pathology and Plant-Microbe Biology, Cornell University, Ithaca, NY 14853, USA; 3 Boyce Thompson Institute for Plant Research, Ithaca, NY 14853, USA; 4 Department of Genome Sciences, University of Washington, Seattle, WA 98109, USA

## Abstract

Virions of the RPV strain of *Cereal yellow dwarf virus-RPV* were purified from infected oat tissue and analysed by MS. Two conserved residues, K147 and K181, in the virus coat protein, were confidently identified to contain epsilon-*N*-acetyl groups. While no functional data are available for K147, K181 lies within an interfacial region critical for virion assembly and stability. The signature immonium ion at *m*/*z* 126.0919 demonstrated the presence of *N*-acetyllysine, and the sequence fragment ions enabled an unambiguous assignment of the epsilon-*N*-acetyl modification on K181. We hypothesize that selection favours acetylation of K181 in a fraction of coat protein monomers to stabilize the capsid by promoting intermonomer salt bridge formation.

*Potato leafroll virus* (PLRV) and the barley/cereal/maize yellow dwarf viruses, members of the *Luteoviridae* and referred to hereafter as luteovirids, are globally important viruses that infect most staple food crops and are model viruses for studies on the regulation of circulative plant virus transmission by aphid vectors (Gray *et al.*, 2014). Luteovirids are icosahedral, non-enveloped, positive sense, RNA viruses that are retained in the phloem of host plants. All but one luteovirid species share a conserved arrangement of three ORFs in the 3′ half of their genome, two of which encode the structural proteins (Miller *et al.*, 1995). The virus capsid consists predominantly of the coat protein (CP) encoded by ORF3 and a minor amount of the readthrough protein (RTP) translated via sporadic readthrough of the CP stop codon (Brault *et al.*, 1995; Cheng *et al.*, 1994; Filichkin *et al.*, 1994; Wang *et al.*, 1995). There is no structure available for any luteovirid, although chemical cross-linking measurements of infectious PLRV virions recently revealed the sites of protein interaction and the interaction topologies between the structural proteins – a first for this family of viruses (Chavez *et al.*, 2012). These measurements correlated well with previous epitope mapping studies of PLRV (Torrance, 1992).

Despite their relatively simple genetic make-up, luteovirids use remarkable subterfuge to ensure their own propagation and spread (Gray *et al.*, 2014). For example, Rochow’s seminal *Science* paper (Rochow, 1970) revealed that mixed infections of luteovirid species can facilitate heterologous encapsidation of one species’ RNA in the virion of another species. Such manipulation can result in an expansion of potential aphid species that can transmit the virus. The RTP acts *in trans* to retain virus in the phloem where it is available to aphids (Peter *et al.*, 2009), a functionality of the virus that defies the central dogma for plant virus cell-to-cell movement whereby all viruses encode a strategy to promote, rather than restrict, intercellular movement among plant tissues. Ultrastructural and functional studies demonstrated that both the CP and RTP regulate virus recognition and transport of the virus through aphid tissues to facilitate transmission between host plants. Although both luteovirid structural proteins clearly play a large role in plant–aphid–virus interactions, very little is known about the biochemical mechanisms that regulate these processes.

Recent studies suggest a role for post-translational modifications in luteovirid biology. Phosphorylation has been shown to be involved in subcellular targeting of luteovirid proteins (Link *et al.*, 2011), in particular the PLRV ORF4 product, P17. P17 targeting to plasmodesmata, the structures that connect plant cells to one another, in *Arabidopsis thaliana* was dependent upon phosphorylation of S71 and S79. This study showed that MS is a powerful tool for mapping post-translational modifications of luteovirid proteins and that such modifications play a role in the biochemistry of the virus. Using less direct methods, treatment of *Turnip yellows virus* (formerly beet western yellows virus) with peptide-*N*-glycosidase F was found to disrupt aphid transmission of the virus, suggesting *N*-linked glycosylation occurs on either the virus structural proteins (Seddas & Boissinot, 2006) or more likely the interacting plant proteins (Revollon *et al.*, 2010). However, the specific roles of these modifications in mediating virus movement and transmission remain ambiguous. Furthermore, there is a paucity of data on post-translational modifications of any luteovirid protein in the context of natural plant infection, where phloem localization is the rule, not the exception.

*Cereal yellow dwarf virus-RPV* (CYDV-RPV) was purified from systemically infected oat tissue (*Avena sativa*, cv. Coast Black) in a two-step process (Fig. 1a); first differential centrifugation was used (Cilia *et al.*, 2012), and then virus was further enriched by immunoprecipitation using a CYDV-RPV-specific polyclonal antibody covalently coupled to magnetic Dynabeads (Invitrogen) (Tamborindeguy *et al.*, 2013). Typical yield for each of three virus preparations prior to immunoprecipitation was between 0.6 and 0.8 mg of virus per kilogram of 4–5 week old, infected plant tissues. All preparations were detected using double antibody sandwich ELISA with anti-RPV antibodies, and each virus preparation was transmissible to plants by the aphid vector *Rhophalosiphum padi* following acquisition from Parafilm membrane feeding sachets as described (Cilia *et al.*, 2012). The immunoprecipitated virus was denatured using 5 mM tris(2-carboxyethyl)phosphine (TCEP) and the cysteine residues were blocked using 10 mM methylmethanethiosulfonate (MMTS). Proteins were digested using modified trypsin (Promega) at a ratio of 1 : 200 at 37 °C overnight according to the manufacturer’s protocol for ProteaseMAX surfactant (Promega) (Fig. 1a). Peptides were dried down and reconstituted in 30 µl buffer A (0.1 % formic acid in water) prior to separation using C18 reversed phase nanoscale liquid chromatography coupled to electrospray ionization. MS analysis was performed in data-dependent mode on an Orbitrap Velos (Thermo Fisher) as described previously (Pinheiro *et al.*, 2014). Raw files were converted to mascot generic format using tools from proteowizard (Kessner *et al.*, 2008) and searched using mascot (Perkins *et al.*, 1999) version 2.3 against a custom database containing, in part, luteovirid sequences (Tamborindeguy *et al.*, 2013). Methylthio- was selected as a fixed modification on cysteines; deamidation of glutamine and asparagine, oxidation of methionine and acetylation of lysine were selected as variable modifications. One missed tryptic cleavage site was permitted, allowing for the identification of one acetylated internal lysine residue per peptide. percolator (Brosch *et al.*, 2009; Spivak *et al.*, 2009) was used to post-filter the spectral matches. The peptide false discovery rate was 0.74 %. The CYDV-RPV CP/RTP had a protein score of more than 24 000 in each of the samples analysed and a mean emPAI value of 120. Sequence coverage of the RTP, including the CP-derived sequence, was between 70 and 74 % in each sample (Fig. 1b). As is typically observed with virus purified from systemically infected tissue, peptides matching the C-terminal domain of the RTP were not detected (Cilia *et al.*, 2012) (Fig. 1b).

**Fig. 1.**
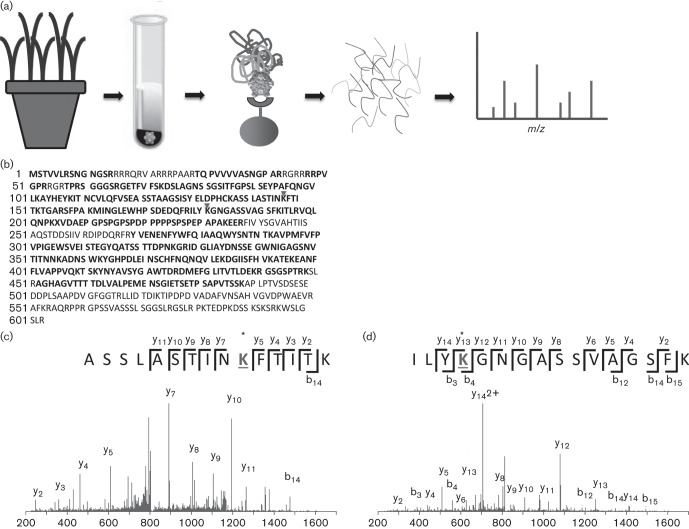
Discovery proteomics workflow identifies acetylated lysine residues in the CP of CYDV-RPV. (a) Oat tissue was homogenized and virions were enriched by density centrifugation followed by immunoprecipitation. The beads were subjected to tryptic digestion and the resulting peptides were analysed by tandem MS. (b) Tryptic coverage of the RTP (including the CP). Acetylated lysine residues are indicated with a triangle above the residue. Tryptic peptides are in bold type. (c) Tandem mass spectrum produced using collision induced dissociation (CID) in the Velos ion trap of the peptide ASSLASTIN_Ac_KFTITK. (d) Tandem mass spectrum of the peptide ILY_Ac_KGNGASSVAGSFK, produced using CID in a Velos ion trap.

Although the sampling and analysis would not specifically enrich for peptides containing acetylated lysine residues, two peptides were detected within the CP that contained acetylated lysine residues (Fig. 1c, d). A peptide spanning CP residues 138–152, K.ASSLASTIN_Ac_KFTITK.T, was detected as a doubly charged precursor ion at *m*/*z* 812.451 8, and had an expect value (*E*-value) of 4.1e^−05^, an ion score of 44 and a mass measurement error of 0.68 p.p.m. No fragmentation was observed around the acetylated lysine residue, K147 (Fig. 1c). A second peptide, spanning CP residues 178–193, R.ILY_Ac_KGNGASSVAGSFK.I, was also detected as a doubly charged precursor ion at *m*/*z* 820.935 7, and had an *E*-value of 5.4e^−06^, an ion score of 53, and a mass measurement error of −0.025 p.p.m. Fragmentation around the acetylated lysine residue, K181, produced both b- and y-ion fragments (b4, y13), containing the acetylK181 (Fig. 1d). Two additional peptides, FTIT_Ac_KTGAR and SFPA_Ac_KMINGLEWHPSDEDQFR, spanning CP residues 148–156 and 157–177, respectively, were also detected with internal acetyllysines on K152 and K161, respectively. However, the *E*-values for these matches were higher at 7.1e^−03^ and 1.2e^−02^. Fully and semi-tryptic variants without acetylation of these internal lysine residues on all four peptides were also confidently identified (not shown) in the datasets, indicating that these lysine residues exist in the capsid in both acetylated and positively charged (containing the ammonium ion) forms. To the best of our knowledge, these data are the first evidence of post-translational modifications of the luteovirid structural proteins and the structural proteins of any non-propagative plant virus. This finding is significant because virions are the vehicles for the movement of the virus genome in plant hosts and insect vectors (Gray *et al.*, 2014).

To glean insights into the conservation of these lysine residues across multiple species of luteovirids, clustal w (Thompson *et al.*, 2002) and WebLogo (Crooks *et al.*, 2004) were used to investigate conserved patterns of amino acid topologies and biophysical properties across twenty virus species spanning the three genera of the *Luteoviridae*. The modified lysines in both peptides are nested within a region that is heavily biased towards polar uncharged and/or hydrophobic residues (Fig. 2). The acetylation of lysine would effectively remove the positively charged ammonium group on the lysine side chain and render the peptide topology neutral in these regions. The conservation of K147 is less than that of K181, which may indicate roles for acetylation of K147 that are specific to the determination of host or vector ranges mediated by specific protein interactions. *Barley yellow dwarf virus* (BYDV) species PAV, PAS, MAV and GAV all have a serine at site K147, and Rose spring dwarf associated virus (RsDAV) and Pea enation mosaic virus (PEMV) have a threonine. These residues could be all modified in such a way as to make the site more or less charged (a phosphate group would add a negative charge, for example). Acetylation of K181 may play a more critical role in luteovirid biology, as described below.

**Fig. 2.**
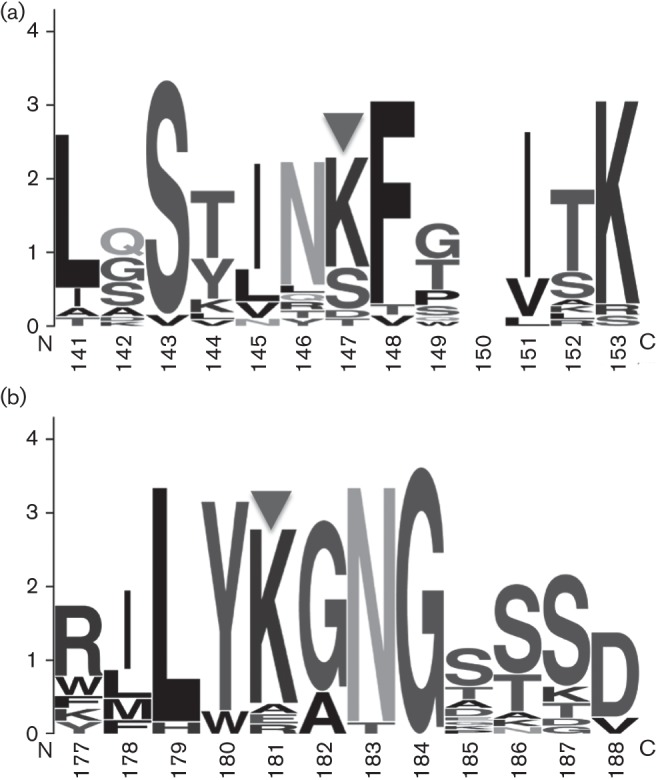
WebLogo alignments for the amino acids surrounding the modified lysine residues in the CYDV-RPV coat protein. Alignments of K.ASSLASTIN_Ac_KFTITK.T (a) and R.ILY_Ac_KGNGASSVAGSFK.I (b), where the residues preceding and following the tryptic cleavage site are included and Ac indicates the acetylated lysine residue. Results were derived from a clustal 2.1 alignment of 20 virus species of all three genera in the *Luteoviridae*. Amino acid residue number on the *x*-axis corresponds to the residue positions of RPV. The height of each stack of residues (for each residue position) indicates the sequence conservation at that position. The height of each single letter within the stack is a measure of the relative frequency of the amino acid at that position (Crooks *et al.*, 2004).

The importance of K181 in luteovirid biology is further supported by evidence from the analysis of virus mutants and studies using Protein Interaction Reporter (PIR) technology. Mutations in the CP and RTP of PLRV, a polerovirus related to CYDV-RPV, can result in phenotypic defects in virus assembly, systemic infection, and aphid transmission (Chavez *et al.*, 2012; Gray *et al.*, 2014; Kaplan *et al.*, 2007; Lee *et al.*, 2005; Peter *et al.*, 2008, 2009). One of these CP mutants, GNG, has three deleted residues, flanking K181. The GNG mutant failed to assemble stable virions *in planta* (Kaplan *et al.*, 2007), demonstrating a critical role for these residues in virion integrity. Furthermore, a peptide spanning these residues was found cross-linked as a homodimer using PIR technology (Chavez *et al.*, 2012). The cross-linked homodimer peptide pair proved that close interactions between CP monomers at the KGNG interface are involved in CP multimer formation and provided structural insight into why this deletion mutant failed to assemble virions. In other biological systems, including a variety of proteins with distinct function and subcellular location, sites of chemical cross-linking coincide with sites of lysine acetylation (Bruce, 2012). In other pathogens, lysine acetylation is commonly associated with regions of high intrinsic disorder (Xue *et al.*, 2013), and such disorder is a hallmark feature of the luteovirid coat protein in this region (Chavez *et al.*, 2012; Gray *et al.*, 2014).

We hypothesize that the acetylation status of K181 regulates virion stability and protein–protein interactions at this critical KGNG interfacial region. For example, acetylation of K181 may serve to stabilize interactions between CP monomers, promoting a disorder-to-order transition that would have a major impact on protein topology in this region of the capsid. Not mutually exclusive to the potential impact on disorder in this region, lysine acetylation has been shown to promote stabilizing salt bridges in other higher-order polymeric proteins (Cueva *et al.*, 2012). In our published PIR-derived PLRV model, the cross-linked K181 residues were approximated to be within 5 Å (0.5 nm). Thus, there is the possibility of stabilizing salt bridges forming within this region. One hypothesis is that acetylation of K181 decreases the probability of intramonomer salt bridge formation and promotes intermonomer salt bridge formation, which results in more uniform packing of the virion. This hypothesis, although just one of many, is consistent with the structural, biochemical and mutational analysis that we have to date.

To provide further support for this hypothesis, residues in this region would have to be available for salt bridge formation. In RPV, the peptide that we found acetylated was also measured to be deamidated at N183 (Fig. 3), which results in a centrally located aspartic acid in this stretch of basic residues. Deamidation of N183 would provide the anionic carboxylate for stabilizing salt bridges to form between it and K181 (or K185 in PLRV) of different monomers. The presence of the glycine residue flanking N183 (Fig. 1d) would enhance the rate of N183 deamidation by an order of magnitude over that theoretically predicted for spontaneous deamidation (Radkiewicz *et al.*, 2001). Intriguingly, the NG dipeptide motif is conserved in all members of the *Luteoviridae* except PEMV (data not shown). Conservation across all these virus species suggests selection pressure on N183 deamidation and for the virus to maintain some flexibility to determine the charge at this site (it is intriguing to note that selection has not produced any luteovirids that simply encode an aspartic acid residue at that position).

**Fig. 3.**
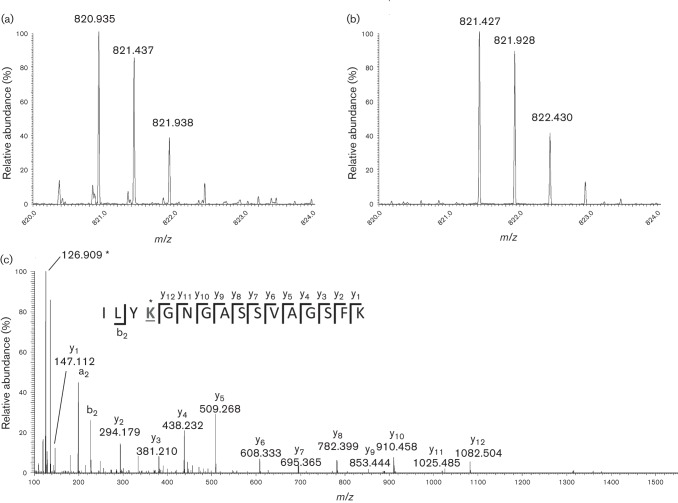
Use of selected ion monitoring (SIM) and high-accuracy/high-resolution measurement of peptide CID fragment ions using an Orbitrap mass analyser. (a, b) SIM scans for both amidated and deamidated peptides (retention times 85.5 and 88 min, respectively). (c) MS/MS spectrum of ILYK_Ac_GDGASSVAGSFK. The *m*/*z* values for the y-ions are labelled, and the acetyllysine-specific fragment ion (Robinson *et al.*, 2004) is indicated by an asterisk. A McLafferty plot shows the positions of each y-ion and the b_2_-ion observed in the peptide. The acetylated lysine residue that produced the immonium ion at 126.089 1 is underlined. The observed mass of *m*/*z* 126.089 1 is 0.002 8 lower than the calculated mass of 126.091 9; however, the immonium ion of tyrosine at *m*/*z* 136.073 3 is off by the same amount (calculated mass 136.076 2). The MS-Product Tool in Protein Prospector (http://prospector.ucsf.edu/prospector/mshome.htm) was used to manually assign the peaks.

To unambiguously assign the deamidation of N183 and acetylation on K181, the sample was reanalysed in such a manner as to specifically target charge and acetylation state variants of this peptide. Each precursor mass was subjected to an *m*/*z* 10 wide selected ion monitoring (SIM) acquisition, followed by collision induced dissociation using the HCD cell on an Orbitrap Velos mass spectrometer and subsequent mass analysis in the Orbitrap analyser. The latter provided high mass accuracy and high-resolution measurement of fragment ions. As this peptide contains the NG sequence which can rapidly deamidate to DG (Robinson *et al.*, 2004) and such deamidation would be required to support the stabilizing salt bridge hypothesis, it was encouraging to observe chromatographic peaks for both peptides, the amidated peptide eluting a couple of minutes prior to the deamidated one. The corresponding SIM scans (range *m*/*z* 816–826) of the amidated and deamidated precursor ions are shown in Fig. 3(a, b), respectively. The MS/MS spectrum of the deamidated peptide, R.ILY_Ac_KGDGASSVAGSFK.I, is shown in Fig. 3(c), which exhibits a contiguous series of y-ions that delineate the sequence GDGASSVAGSFK, and also contains the signature epsilon-*N*-acetyllysine immonium ion at *m*/*z* 126.090. These data unambiguously assign an acetylation site to K181 (Trelle & Jensen, 2008).

Protein acetylation has received widespread attention in animal biology (Glozak *et al.*, 2005; Li *et al.*, 2012; Yang *et al.*, 2011) and in mediating RNA virus infection in animals (D’Orso & Frankel, 2009; Mu *et al.*, 2004). Genome sequencing has revealed a variety of acetyltransferases and deacetylases in a variety of plant genomes, including monocot cereals (Nallamilli *et al.*, 2014). Overall, less is known about lysine acetylation in plants (Wu *et al.*, 2011), although acetyl-coenzyme A (acetyl-CoA) is a central metabolite involved in most plant biochemical pathways as well as in protein acetylation (Xing & Poirier, 2012). Acetyl-CoA is a key molecule linking cellular metabolism and protein function via acetylation in plants (Xing & Poirier, 2012). Quite intriguingly, a major protein differentially expressed in both lab-reared and naturally occurring vector and non-vector populations of *Schizaphis graminum*, a vector species for CYDV-RPV, is an acetyl-CoA ligase (Cilia *et al.*, 2011a, b; Yang *et al.*, 2008). Those data suggest that *S. graminum* CoA ligase may play a central role in determining the availability of acetyl groups to viral or aphid protein substrates and that regulation of the acetylation helps to determine variation in vectoring capacity in this species. These new data suggest that further studies on the role of acetylation of luteovirids in virus assembly, host systemic infection and circulative transmission are pertinent.
